# Genomic Heterogeneity of Osteosarcoma - Shift from Single Candidates to Functional Modules

**DOI:** 10.1371/journal.pone.0123082

**Published:** 2015-04-07

**Authors:** Kathrin Poos, Jan Smida, Doris Maugg, Gertrud Eckstein, Daniel Baumhoer, Michaela Nathrath, Eberhard Korsching

**Affiliations:** 1 Institute of Bioinformatics, University Hospital Münster, Münster, Germany; 2 Clinical Cooperation Group Osteosarcoma, Helmholtz Zentrum München, German Research Center for Environmental Health, Neuherberg, Germany; 3 Children's Cancer Research Center and Department of Pediatrics, Klinikum rechts der Isar, Technische Universität München, Munich, Germany; 4 Institute of Human Genetics, Helmholtz Zentrum München, German Research Center for Environmental Health, Neuherberg, Germany; 5 Bone Tumor Reference Center at the Institute of Pathology, University Hospital Basel, Basel, Switzerland; Princess Margaret Cancer Centre, CANADA

## Abstract

Osteosarcoma (OS), a bone tumor, exhibit a complex karyotype. On the genomic level a highly variable degree of alterations in nearly all chromosomal regions and between individual tumors is observable. This hampers the identification of common drivers in OS biology. To identify the common molecular mechanisms involved in the maintenance of OS, we follow the hypothesis that all the copy number-associated differences between the patients are intercepted on the level of the functional modules. The implementation is based on a network approach utilizing copy number associated genes in OS, paired expression data and protein interaction data. The resulting functional modules of tightly connected genes were interpreted regarding their biological functions in OS and their potential prognostic significance. We identified an osteosarcoma network assembling well-known and lesser-known candidates. The derived network shows a significant connectivity and modularity suggesting that the genes affected by the heterogeneous genetic alterations share the same biological context. The network modules participate in several critical aspects of cancer biology like DNA damage response, cell growth, and cell motility which is in line with the hypothesis of specifically deregulated but functional modules in cancer. Further, we could deduce genes with possible prognostic significance in OS for further investigation (e.g. *EZR*, *CDKN2A*, *MAP3K5)*. Several of those module genes were located on chromosome 6q. The given systems biological approach provides evidence that heterogeneity on the genomic and expression level is ordered by the biological system on the level of the functional modules. Different genomic aberrations are pointing to the same cellular network vicinity to form vital, but already neoplastically altered, functional modules maintaining OS. This observation, exemplarily now shown for OS, has been under discussion already for a longer time, but often in a hypothetical manner, and can here be exemplified for OS.

## Background

Osteosarcoma (OS) is characterized by neoplastic cells that directly produce immature osteoid [1, 2]. It exhibits a complex karyotype resulting from high rates of genomic instability, in particular chromosomal instability [3]. Several inherited cancer susceptibility diseases are related to OS like the Li-Fraumeni syndrome (*TP53* germline mutation) [4], the Retinoblastoma (*RB1* germline mutation) [5], or the Werner syndrome (*WRN* germline mutation) [6]. These familial syndromes are rare and do not represent a common cause of OS. However, they affect genes that are responsible to maintain genome integrity and therewith provide a link to chromosomal instability [3, 7].

To address chromosomal instability underlying OS, many studies showed genomic alterations and suggested potential candidate genes driving OS development [3, 8]. In various regions, one can observe gains and losses of entire chromosomes or chromosomal segments. Many oncogenes and tumor suppressor genes are located within these sites [3, 7]. Frequently observed genomic gains contain the chromosome arms 6p, 8q, and 17p that include oncogenes like *MYC* and *COPS3* [9–12]. Recurrent regions of losses involve chromosome arms 3q, 9p, 13q, and 17p containing tumor suppressor genes like *LSAMP*, *CDKN2A*, *RB1*, and *TP53* [8, 10, 13, 14]. Various other genomic changes and altered genes have been reported. Some of them are implicated in mitotic checkpoint control, whose deregulation is assumed to be the underlying cause of chromosomal instability [3]. However, there is a wide range of reported alterations and a common effect has not yet been identified [3, 7]. Despite the information of many genetic changes, OS is only defined by its morphological and clinical phenotype rather than on the molecular level [15]. This inter-tumor heterogeneity might be formalized by integrating copy number associated genes on the biological network-level. Cellular functions within biological networks are thought to be carried out in a modular manner. Individual modules consist of highly connected nodes such as genes or proteins that act together in the same functional context [16]. Cerami et al. [17] developed a systems biological approach to uncover altered network modules in glioblastoma. They showed that different combinations of altered genes can prevent modules to perform their natural biological function. Further, they stated that glioblastoma development occurs via different genes and diverse mechanisms but within the same functional modules. According to these findings, OS might develop primarily due to heavily accumulated genomic alterations secondarily causing the inability of genes within distinct modules to perform their normal biological functions. Hence, we might observe heterogeneity on the gene-level but a distinct set of functional modules on the network level.

**Fig 1:**
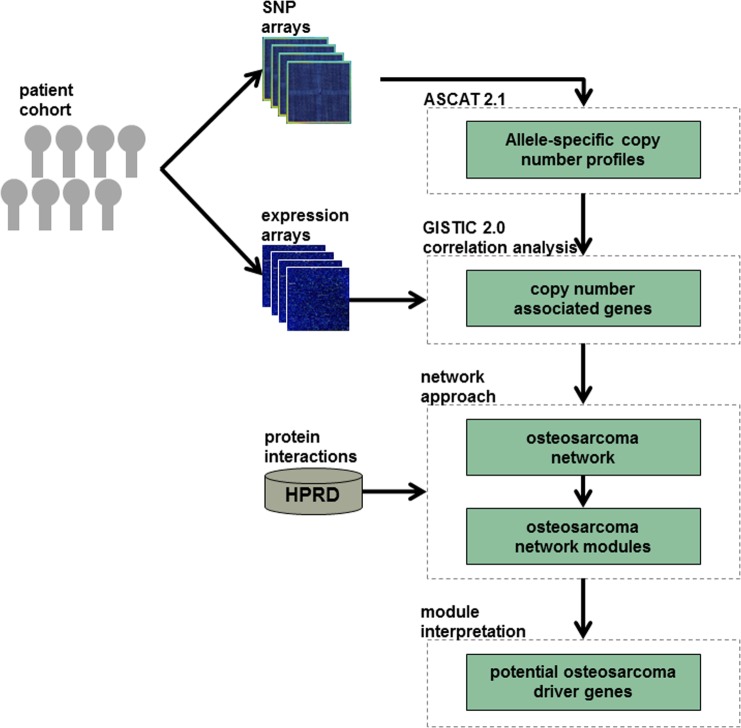
Analysis workflow. The figure gives an outline of the analysis performed in the current study.

In this study, we investigated the enrichment of copy number associated genes within cellular modules in OS and also give some preliminary insight on their impact on patients’ survival. On that account, we analyzed paired copy number and expression data derived from a series of 44 pre-therapeutic OS biopsies. First, allele-specific copy number profiles were determined by considering tumor ploidy and the non-aberrant cell fraction within the tumor tissue. Therefrom, we defined significant gained and lost regions. The copy number profiles of these regions were correlated with expression data to obtain copy number associated genes in OS. Next, we mapped the copy number associated genes on protein interaction data and constructed an OS network. This network was analyzed regarding its module structure and functional implications in OS development and prognosis (Fig 1). The results point towards the value of systems biological approaches and to the need to extend the classical driver gene hypothesis to a more appropriate 'functional module' hypothesis to understand OS biology.

## Methods

Some of our terms, and how we use them throughout the text, are explained in Table 1. The RMA normalized and gene based expression data, the R source code and the module visualization in Cytoscape can be obtained from GitHub https://github.com/korpleul/PONED1451866R1.

**Table 1:** Definition of used terms.

driver genes	Genes within the network that are copy number altered according to GISTIC. These genes are thought to play a specific role in OS. Those genes are further filtered by thresholds and the correlation with expression values.
linker genes	Genes that connect the copy number altered driver Genes to a network. Copy number altered driver genes which do not belong to the network due to a missing direct connection in that network might be connected by an additional gene ('first neighbor nodes'). This process integrates isolated copy number altered driver genes to a functional module. The biological meaning has to be checked by a priory knowledge.
hub genes	Genes that are highly connected (see connectivity) within the global network or within network modules. Those genes might coordinate different biological pathways.
functional module	Subnetwork from the global network, that contains genes more connected to each other that to genes of the global network. Module genes are likely to perform the same biological functions.
highly connected	A gene that interact with many other genes in the network.
edge betweenness	Algorithm to define highly connected gene modules.
connectivity	Number of interactions one gene has within the network (similar to node degree).
cellular network vicinity	Genes that directly interact to each other build a neighborhood (in a functional sense).

Further readings e.g.: Barabasi AL, Oltvai ZN. Nat Rev Genet. 2004 Feb;5(2):101–13. Network biology: understanding the cell's functional organization.

### Tissue samples and patient characteristics

A series of 44 fresh-frozen, pre-treatment OS tissue samples was selected for this study. The samples were collected between 1992 and 2007 (Cooperative German-Austria-Swiss Osteosarcoma Study Group). We conducted this study according to the principles expressed in the Declaration of Helsinki. The patient cohort samples used in this study were obtained according to the guidelines and approval of the Faculty of Medicine of the Technical University of Munich Research Ethics Board (Technische Universität München TUM, Reference 1867/07) and local ethical committee of Basel, Switzerland (Ethikkommission beider Basel EKBB, www.ekbb.ch, Reference 274/12). Informed written consent to participate in this study was obtained from the patients, or in the case of young children, their next of kin, caretakers, or guardians on their behalf. Out of 12 patients, who developed metastasis, 7 showed metastasis at the time of diagnosis. Pre- and postoperative chemotherapy was applied according to the protocols of the Cooperative Osteosarcoma study (COSS) group. Response to chemotherapy was classified according to the Salzer-Kuntschik (SK) histological grading system [18]. Good responders (SK grades 1 to 3) exhibited ≤ 10% viable tumor cells and bad responders (SK grades 4 to 6) showed >10% viable tumor cells following neoadjuvant chemotherapy. SK grades were available for 35 patients. Follow-up data were available for all 39 patients (15 females and 24 males with an age span from 4 to 60 years and median of 15 years) with mean follow-up time of 69 months ranging from 5 to 185 months. These 39 were used for the survival analysis. Further patient characteristics are given in Table 2.

**Table 2:** Clinicopathological patient characteristics .

Characteristics	# patients (n = 39)
**Age at diagnosis (years)**	
mean	19
median	15
range	4 to 60
**Gender**	
male	24
female	15
**Tumor localisation**	
femur	21
tibia	7
fibula	2
sacrum	2
inguinal	1
knee	1
lower leg	1
pelvis	1
scapula	1
second metatarsal	1
ulna	1
**Histological subtype**	
osteoblastic	22
osteoblastic+chondroblastic	5
cellular	3
chondroblastic	3
none	2
fibroblastic	1
giant cell rich	1
small cell	1
unknown	1
**Metastasis**	**12**
metastasis at diagnosis	7
**Response to chemotherapy** ^**a**^	**35**
good	18
SK I	0
SK II	10
SK III	8
poor	17
SK IV	10
SK V	5
SK VI	2
**Clinical outcome** ^**b**^	
CR1	27
DOD	9
AWD-LOFU	1
CR1-LOFU	2
**Follow-up months**	
mean	69
median	71
range	5 to 185

^a^ The Salzer-Kuntschik (SK) grading system provides six grades: SK I—no residual viable tumor, SK II—solitary viable tumor cells; SK III—< 10% viable tumor cells; SK IV—10 to 50% viable tumor cells, SK V—50 to 80% viable tumor cells; and SK VI—> 80% viable tumor cells.

^b^ CR—complete remission; DOD—dead of disease; AWD—alive with disease; LOFU—lost to follow up.

### Copy number data analysis

Affymetrix’s Genome-Wide SNP arrays 6.0 were used for copy number data analysis (Affymetrix Inc.). The data is publicly available in the ArrayExpress database (www.ebi.ac.uk/arrayexpress) under accession number E-MTAB-3034 and by Dr. Jan Smida on behalf of the Clinical Cooperation Group Osteosarcoma, Helmholtz Zentrum München, German Research Center for Environmental Health, Neuherberg, Germany (smida@helmholtz-muenchen.de). DNA samples were processed due to the manufacturer’s recommendations. To transform raw data to LogR ratios and B-allele frequencies for further copy number detection, we run PennCNV-Affy (http://www.openbioinformatics.org/penncnv/penncnv_tutorial_affy_gw6.html) [19]. Processed genotype data were further analyzed for allele specific copy numbers using the ASPCF segmentation algorithm (ASCAT version 2.1) [20]. ASCAT estimates and corrects segmented genotype data derived from tumor samples, for tumor ploidy and non-aberrant cell fraction, to obtain allele-specific copy number profiles. In this study, ASCAT failed to process 3 out of 44 samples so 41 go into the network analysis. We additionally filtered for copy number segments overlapping (>50%) with telomeric or centromeric regions and segmental duplications. Human genome information was downloaded from the UCSC genome table browser (assembly hg19) [21]. The frequency of gains and losses was determined relative to tumor ploidy. If the copy number was more or less than 0.9 above or below tumor ploidy, we called the SNP gained or lost, respectively. A minimum number of 10 consecutively aberrant SNP markers were required to call regions of gains and losses. Only regions consistently altered in at least 20% of all samples were considered as recurrent regions of copy number alteration. Significantly gained or lost regions in the genome, among the OS samples, were determined using GISTIC 2.0 [22]. It was run by entering the ASPCF segmented LogR ratios. Segments with a LogR ratio of +/- 0.12 were called gained or lost. We chose here a less stringent value to conserve the genomic phenotype of OS. The value is also justified by matrix CGH experience [23] and by considerations in Mermel et al. [22]. Additionally the concept of segmentation itself is limited in its resolution, and we own here a highly variable genome. Therefore the established algorithms might not be seen as a real sensitive solution to this situation which also justifies the less stringent value. 10 consecutively aberrant SNP markers were required to call significantly gained or lost regions.

### Gene expression data analysis

Gene expression data of the same OS samples were obtained using the Human Gene 1.0 ST array from Affymetrix (Affymetrix Inc.). The data is available by Dr. Jan Smida on behalf of the Clinical Cooperation Group Osteosarcoma, Helmholtz Zentrum München, German Research Center for Environmental Health, Neuherberg, Germany (smida@helmholtz-muenchen.de). Prior to data pre-processing, RNA was isolated and further processed as described in [24]. Data pre-processing was done using the Bioconductor package affy [25]. The raw probe intensities were background corrected, normalized, and summarized to the gene-level by applying the robust multi-array average algorithm (rma) [26].

### Detection of copy number associated genes

To evaluate whether expression of genes located within statistically significant genomic alterations was copy number associated, we superimposed (paired) gene expression and copy number data, and calculated the Pearson correlation coefficient. To assess the significance of the correlation coefficients, a null distribution was generated based on random permutation across the samples. We performed 1,000 random permutations and defined the sampling p-value as: *sum (correlation coef*. _*random*_ *> correlation coef*.*) / number*_*permutations*_. The resulting p-values were corrected for multiple testing (False Discovery Rate, FDR <0.1) [27]. So the resulting genes were associated with copy number variable regions and a conforming expression characteristic.

### Network approach to identify osteosarcoma drivers

We adapted the network analysis from Cerami et al. [17] to identify enriched modules in OS with the statistical computing environment R [23, 28] using the Bioconductor packages graph [29] and igraph [30]. To determine the network of copy number altered genes in OS, protein interaction data derived from the Human Protein Reference Database (HPRD) version 9 [31] was used. Following, when referring to edges of a network, we implicitly mean gene- and protein interactions. These words are used synonymously throughout this study.

#### Identification of an osteosarcoma network

The OS network, including the significant linker genes, was identified by superimposing the previously selected significant genes on to the HPRD and by selecting their first neighbor nodes. The neighboring nodes, connecting at least two significant genes, were kept in further analysis and called linkers. We tested, if the linker nodes connected more copy number altered genes in OS than expected by chance. Therefore, their number of neighbors in the HPRD was compared to the number of interaction partners within the OS network using the hypergeometric test. The resulting linker nodes were filtered by FDR <0.05. Further, the connectedness of the derived OS network was tested against randomly selected networks. 449 copy number associated genes were present in the HPRD. Hence, we selected the same number of genes for a random network generation. We sampled 1,000 times and selected at every time all genes respective linker nodes present in the HPRD. Further, we compared the number of nodes and edges of the random networks with the observed one and computed empirical p-values for the OS network’s connectedness. The sampling p-values were determined by *sum (connectedness*_*random*_ *> connectedness) / number*_*permutations*_.

#### Determination of osteosarcoma network modules

To determine closely connected modules within the derived OS network, we run the edge betweenness algorithm and assessed its modularity score [32]. Statistical significance of the network’s modularity was evaluated by the edge swapping algorithm [33]. We generated 1,000 random networks of fixed size and node degrees as the observed OS network. For each swapped network, the modularity was computed and compared to the observed network using the scaled modularity score [34].

### Network visualization, module annotation, and survival analysis

The biological network respectively the modules were visualized using Cytoscape [35]. Nodes in the network represent genes implicated in OS development. They are color coded according to the type of genomic alteration: green for copy number losses, red for copy number gains, and gray for linker genes. The size of nodes corresponds to the number of tumor samples with a distinct gene alteration. Altered genes are represented as circles and linker genes as diamonds. To analyze the network topology, we computed the node degree distribution. The node degree is defined as the number of direct neighbors of a node in a network. Nodes having a high number of direct neighbors are thought to be important regulatory hubs inside the network [16]. Individual network modules were functionally annotated using the Gene Ontology (GO) enrichment analysis from Bioconductor’s GOStats package [36]. Enrichment was tested against all genes of the HPRD network (background set). Multiple test correction was performed using the FDR approach [27]. The survival curves were generated using the Kaplan-Meier method, and a log-rank test was applied to determine the prognostic significance by using the R package survival [37].

## Results and Discussion

### Representativeness of tumor samples and reliability of ASCAT approach

Copy number data of 44 fresh-frozen, pre-treatment tissue samples from OS patients were achieved using Affymetrix SNP 6.0 arrays. As tumor samples comprise different cell populations of tumor and non-tumor cells, and often deviate from the diploid state, it is necessary to correct copy number profiles for tumor ploidy and aberrant cell fraction [20]. This has been shown to be true for OS [38], too. Running ASCAT resulted in allele-specific copy number profiles corrected for intra-tumor heterogeneity. Out of 44 samples, ASCAT failed to predict copy number profiles of 3 tumor biopsies that were excluded from further analyses. According to ASCAT, the tumor biopsies were infiltrated with 31% non-aberrant cells on average with a mean ploidy of 2.8n. No correlation between aberrant cell fraction and tumor ploidy, and occurrence of metastases, or response to chemotherapy was found. However, OS patients whose tumors exhibited a ploidy pattern greater than 3 showed a poorer survival (Chi-square p = 0.053, S1 Fig). This result is in accordance with studies from Kusuzaki et al. who revealed that DNA ploidy patterns are of prognostic significance in OS [39, 40].

After correcting the copy number profiles for aberrant cell fraction and tumor ploidy, we determined genomic gains and losses within our OS series. The number of numerical aberrations between individual patient samples is highly variable. Copy number gains are ranging from 7 to 190 and losses from 7 to 170 per sample, indicating the heterogeneity of OS between different patients. Recurrent copy number variable regions are shown in S2 Fig Prominent gains are located on chromosome arms 6p, 8q/9p and 17p and the losses on chromosome arms 3q, 6q, 8p/9p, 11p, 15q, and 17p among others. Several of these regions have frequently been reported to be gained or lost in different studies of OS [3, 10, 13, 41–45]. The most common gained and lost regions occurred in approximately 34% (8q) and 39% (1p) of OS samples, respectively. Frequently both changes appear jointly. But this relatively low frequency illustrates the diversity of the individual tumors and hampers the identification of common driver genes.

These results are in accordance with already published data demonstrating the quality and representativeness of tumor samples used in the current study. Summing up, **(1)** we also observed a prognostic tendency between tumor ploidy and disease outcome, **(2)** we noticed high variability of chromosomal instability and affected genomic regions between different patients, and **(3)** we determined recurrent regions of genomic alterations also found in other studies.

### Selecting driver genes of osteosarcoma

**Fig 2:**
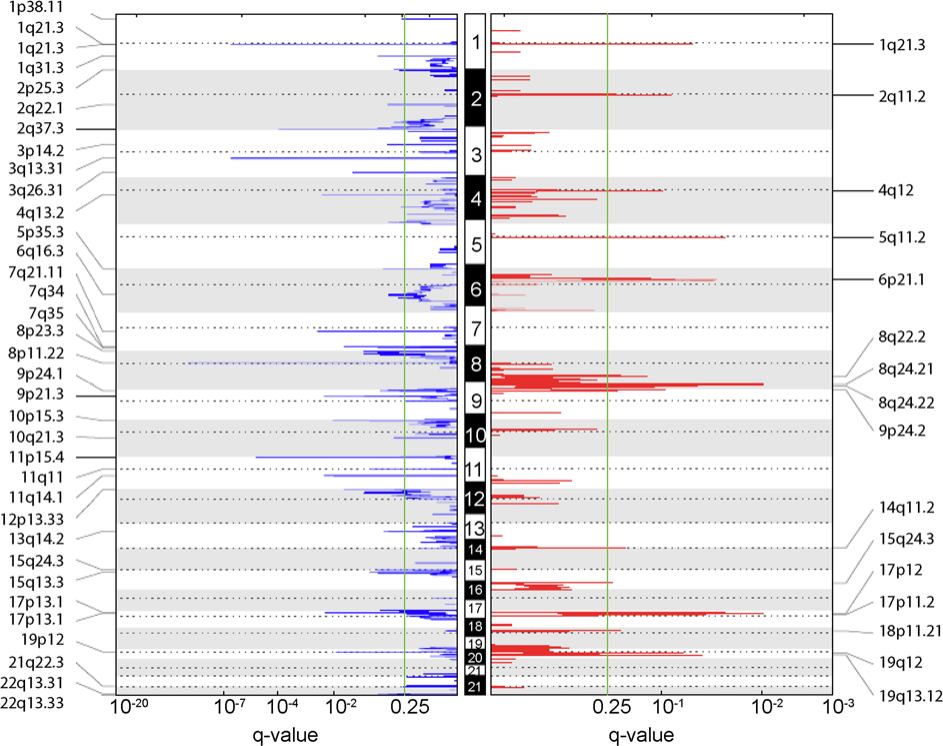
Significant regions of copy number variation in osteosarcoma. The plots show the q-values (x-axes) determined by GISTIC 2.0 with respect to significant lost (blue) and gained (red) genomic regions among the human chromosomes 1 to 22 (hg19). The green lines indicate the significance threshold of q-value <0.25. The cytobands of significant aberrant regions are denoted on the y-axes.

To detect genes located within significant regions of genomic alterations within the OS samples, we run GISTIC 2.0 [22]. GISTIC assesses the significance of numerical aberrations compared to random background and has been shown to detect likely drivers in malignant tumors. The analysis obtained 16 significant copy number gains and 35 significant losses containing 2,392 genes in total (q-value <0.25, Fig 2). The given characteristics is comparable to Both et al. [46] even so they use a different workflow. S1 Table lists the GISTIC results in detail. To explore copy number associated genes within the GISTIC regions, we assessed the quantitative relationship between gene expression derived from Affymetrix Human Gene 1.0 ST arrays and copy number profiles of the same OS samples. We could map the expression values of 1,360 genes located within significant regions of aberrations to their corresponding copy number values. To test weather this result can be also generated by chance, we compared this correlation distribution against the background distribution and also to a distribution based on the recurrent regions. The correlation of the GISTIC genes is significantly higher compared to the background distribution of all genes and to genes located within recurrent genomic alterations (S3 Fig). This result additionally justifies the set of copy number associated genes within GISTIC regions. Genes within the GISTIC regions are affected more directly by genomic alterations than genes located within recurrent regions defined solely by a copy number approach (see previous chapter).

For the further downstream analysis, only genes within GISTIC regions showing significant correlation coefficients, compared to random correlations based on sample label permutations (FDR <0.1), were considered to be copy number associated. In total, 826 genes showed significant correlation values between expression and copy number profiles.

Copy number associated genes contained several prominent players of OS development, e.g. *RB1*, *CDKN2A*, and *CCNE3* [43–45]. However, many candidates frequently mentioned in the context of OS like *TP53*, *CDKN1A*, or *CDK4* were missing [45, 46, 47]. This does not mean that these candidates are not altered or not involved, but point to a heterogeneous situation between the investigated samples concerning their genomic variations and corresponding expression levels (see also S2 Table). Again, this underlines the lack of a strict and common genetic root.

### Enhancing the gene set by a network approach

Up to this point, we have primarily characterized and integrated two data levels. The significance was demonstrated by GISTIC and a second sampling approach. The existing driver gene set might be closer to a OS consensus by adding network or interaction information thus excluding side-effects from the gene list. We did that by adopting a network approach according to Cerami et al. [17]. The OS network was generated by mapping the copy number-associated genes on the HPRD protein interaction data. We connected as much as possible of these genes with each other via direct interactions and via linker genes. I.e., for each copy number associated gene pair, every interaction path of length 1 or 2 was identified. An interaction path of length 1 denotes a direct interaction between two copy number associated genes. A path of length 2 marks two copy number associated genes connected via a linker gene. A gene pair can be connected by multiple linker genes. The linker genes should provide a broader biological context for later module identification and interpretation than purely copy number associated genes [17]. Only linker genes, whose first network neighborhood was significantly (FDR <0.05) enriched for copy number associated genes, were considered for OS network generation. In total, we joined 254 copy number associated genes with each other and 247 linker genes to one connected component. Further, the global connectivity of the copy number-derived OS network was assessed by comparing the observed number of nodes and edges to networks obtained from randomly selected genes from the HPRD (S4 Fig). The OS network is highly connected indicating a similar biological context for the copy number associated genes.

According to the node degree distribution of the OS network (S5 Fig), we defined global hub genes as the top 5% of genes showing the highest number of interactions. Hub genes are highly connected genes within a gene network that are thought to perform crucial functions in the cellular system [16]. Table 3 lists all hub genes of the OS network. In total 28 hub genes are listed. Some of them represent linker genes like *AR*, *CDKN1A*, and *TP53*. According to our osteosarcoma knowledge database (http://osteosarcoma-db.uni-muenster.de/, [47]), almost all of these linker hubs have been implicated in OS development. On the contrary, a few copy number associated hub genes are lesser-known players in OS. However, they were reported in other cancer entities. For instance, *FYN* is implicated in the metastasis of pancreatic cancer [48] and *EEF1A1* plays a role in metastatic progression of prostate cancer [49].

**Table 3:** Global hub genes within the osteosarcoma network.

Entrez geneid	Symbol	Degree	Copy number alteration	Osteosarcoma database
2099	ESR1	47	yes	yes
5925	RB1	41	yes	yes
6908	TBP	33	yes	no
4093	SMAD9	27	yes	yes
5599	MAPK8	22	yes	yes
4188	MDFI	21	yes	no
7157	TP53	20	no	yes
7532	YWHAG	20	no	no
5601	MAPK9	19	yes	yes
2534	FYN	18	yes	no
1915	EEF1A1	15	yes	no
1029	CDKN2A	15	yes	yes
367	AR	13	no	yes
4089	SMAD4	13	no	yes
2963	GTF2F2	13	yes	no
3146	HMGB1	13	yes	yes
6722	SRF	13	yes	yes
3066	HDAC2	12	yes	yes
5970	RELA	11	no	yes
4217	MAP3K5	11	yes	yes
3480	IGF1R	11	yes	yes
55090	MED9	10	yes	no
5592	PRKG1	10	yes	no
6256	RXRA	9	no	yes
1026	CDKN1A	9	no	yes
6885	MAP3K7	9	yes	no
7337	UBE3A	9	yes	no
8773	SNAP23	9	yes	no

The table lists the top 5% of genes with the highest number of interactions in the OS network. For each hub gene the Entrez geneid, the official gene symbol and its node degree is given. The next column shows if the hub gene is copy number-associated while the last column denote if the gene is part of the Osteosarcoma Database (http://osteosarcoma-db.uni-muenster.de/).

The results indicate that independent of the individual OS’s genomic complexity genomic alterations occur within the same cellular network vicinity.

### The network modules depict the cancer biology of OS

The OS network was further analyzed considering its structure and functional implications regarding cancer biology. On that account, we determined the modularity structure of the network. A module is a more densely connected set of genes within the entire network. The members of a module are thought to work in the same functional relationships [16]. We identified 26 modules within the OS network (Table 4). Furthermore, the modularity score of the clustered OS network was compared to the modularity scores of 1,000 rewired networks of fixed size and node degree distribution (S6 Fig). The test revealed that the observed OS network is significantly more modular than expected by chance denoting distinct functional implications in OS development. Next, we computed enriched functional categories for each network module. The most high ranking and informative GO terms were used for module annotation (Table 4).

**Table 4:** Osteosarcoma network modules.

Module	Entrez geneid	Symbol	Hub degree	# nodes	# edges	Biological context
1	5599	MAPK8	17	41	64	MAPK cascade
2	2534	FYN	13	39	44	cell adhesion
3	2099	ESR1	29	92	181	transcription regulation / proliferation
4	4093	SMAD9	16	40	44	chromatin silencing
5	7337/4734	UBE3A/NEDD4	5	11	13	proteolysis
6	7157	TP53	7	12	11	DNA repair
7	28514	DLL1	4	16	15	Notch signaling pathway
8	5592	PRKG1	7	28	33	cell communication
9	8773	SNAP23	8	30	37	membrane fusion
10	26258	BLOC1S6	4	13	13	mitosis
11	9444/57135	QKI/DAZ4	2	4	3	cell differentiation
12	23654/5923	PLXNB2/RASGRF1	3	12	11	GTP metabolic process
13	4089	SMAD4	7	22	24	cell-cell junction organization
14	29127	RACGAP1	2	3	2	cytokinesis
15	701	BUB1B	3	6	5	mitotic spindle checkpoint
16	55090	MED9	8	11	14	transcription
17	1029	CDKN2A	12	27	35	DNA replication
18	5045	FURIN	3	9	8	hormone metabolic process
19	4188	MDFI	12	14	16	macroautophagy
20	6500	SKP1	4	16	17	response to stimulus
21	5689	PSMB1	3	4	4	DNA damage response
22	79873	NUDT18	3	8	7	---
23	7532	YWHAG	12	26	25	G2/M transition of mitotic cell cycle
24	51678	MPP6	4	7	7	rRNA metabolic process
25	84466	MEGF10	4	7	6	chromatin assembly
26	9421	HAND1	2	3	2	---

For each module the Entrez geneid, the official gene symbol, and the hub gene degree for its hub gene is given. Moreover, the number of nodes and edges and the biological context of its members are mentioned.

**Fig 3:**
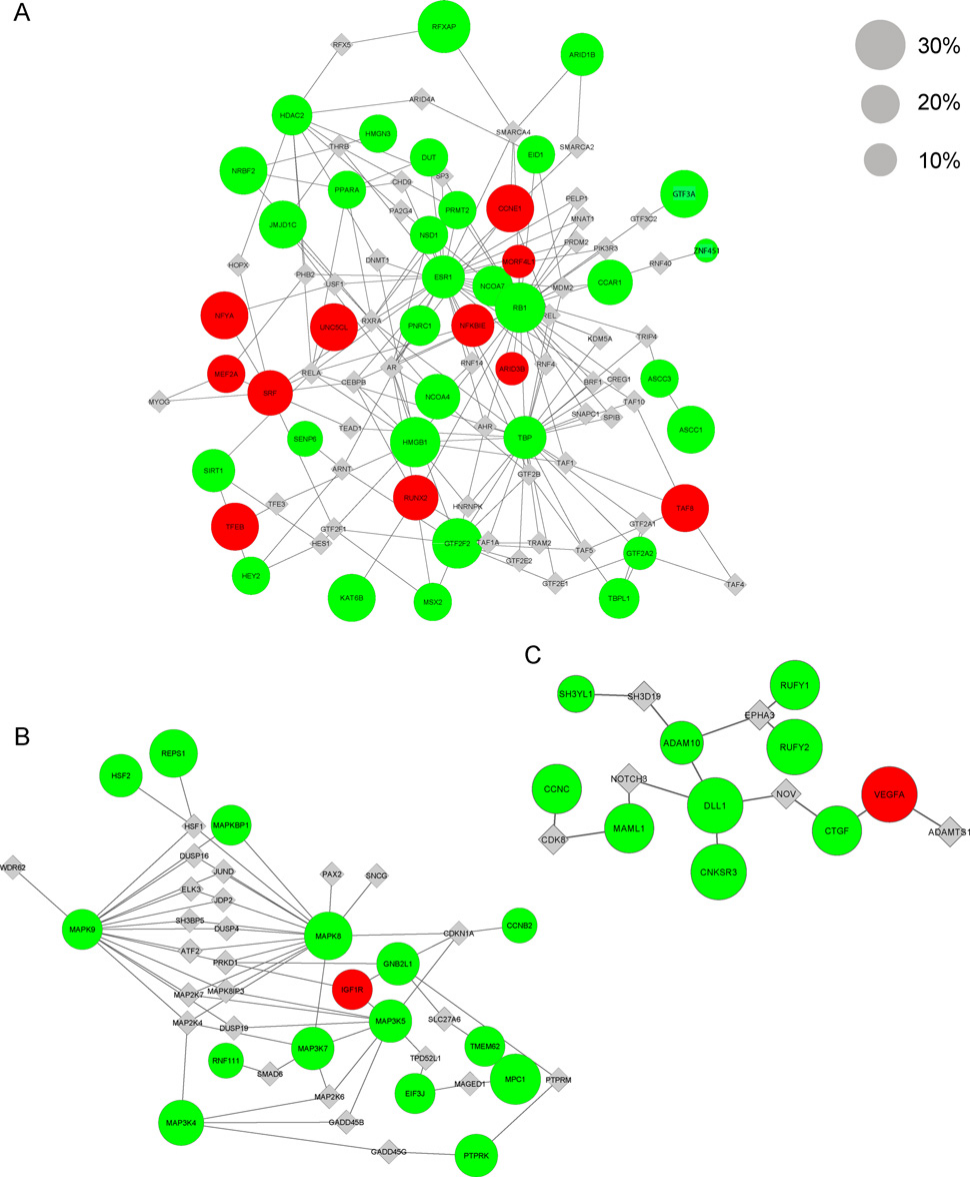
Selected osteosarcoma network modules. In this figures the OS network modules are illustrated for **(A)** the proliferation module 3, **(B)** the MAPK signaling module 1, and **(C)** the NOTCH signaling module 7. They are shown as graphs with nodes and edges. Nodes correspond to genes and edges to their protein interactions derived from the HPRD. Copy number-associated genes are presented as circles and linker genes as diamonds. The size of the nodes corresponds to the percent of OS samples holding a specific copy number loss (green) or gain (red). The linker genes are not altered in OS, therefore they have one size. Cytoband information can be found in Supporting Information S3 Table.

One prominent module is represented by module 3 containing amongst others the *RB1* gene (Fig 3A, S3 Table). It is implicated in transcription and proliferation. The transcriptional program is usually deregulated in cancer, which results in repression of differentiation related genes or activation of oncogenes [50]. Module 3 contains well known transcription factors deregulated in OS. For example, the transcription factor encoded by the gene *RUNX2* (gained) is associated with osteoblast differentiation and OS [51]. The sex steroid receptors *ESR1* (lost) and *AR* (linker) have been reported to be related to OS proliferation [52], which is not wondering as OS frequently affects children and adolescents in times of hormonal changes [7]. Moreover, the members of NFKB signaling, *NFKBIE* (gained) and *RELA* (linker), have been reported to be frequently gained in OS [53] and implicated in OS cell proliferation [54]. To summarize, the mentioned transcription factors pointing to the regulation of cell proliferation, which is one major function of the *RB1* gene product (lost) [55] and its cell cycle members (e.g. *CCNE1* (gained), *CCAR1* (lost), *MDM2* (linker), *TAF1* (linker), *MNAT1* (linker), *SMARCA4* (linker)). Independently from our approach we also annotated module 3 by means of the String database [56]. The String database can be seen as a superset to HPRD, but is used here primarily to illustrate the different known interaction resources for proliferation module 3 (S7 Fig).

Moreover, module 1 and 7 contain members of MAPK and NOTCH signaling as denoted by the hub genes *MAPK8* (lost) and *DLL1* (lost) a NOTCH ligand (Fig 3B and 3C, S3 Table). Both pathways are deregulated in OS development [57, 58]. Further modules are related to DNA damage, stress response, epigenetic processes, mitosis, and cell motility functions essential for tumorigenesis [59]. These modules include OS-associated genes like *CDKN2A* (lost), *TP53* (linker), *EZR* (lost), *FAS* (linker), *WRN* (linker), *HDAC2* (lost) that are involved in the mentioned processes [60–65].

The Cytoscape session of the computed OS network modules is available for further exploration (https://github.com/korpleul/PONED1451866R1). The presented modules illustrate how the molecular factors within the OS network perform distinct functions that commonly define tumor biology.

### Make use of the osteosarcoma network modules

By analyzing individual modules for clinical relevance, we identified gene alterations that might be of prognostic significance. The selection of interesting genes in all modules was primarily focused on the gene members’ topology and different combinations of highly connected genes and their effect on patients survival. Because of the limited amount of patients in our study those first results are of preliminary nature but nevertheless might inspire and stimulate discussion (Table 5). We detected a trend for poor outcome (Chi-square p-value <0.05) for the deleted genes *MAPK9* and *MAP3K5* (module 1), *EZR* (module 2), *EEF1A1* (module 4), *UBE3A* (module 5), *DLL1* and *ADAM10* (module 7), *TCP1* (module 15), *CDKN2A* (module 17), *IGF2R* (module 18), *ANXA11* (module 20), and *SHPRH* (module 23). The deletion of those genes seems to be correlated with poor survival (see S8 Fig for overall survival). In fact, *MAPK9*, *MAP3K5*, *EZR*, *CDKN2A*, and *IGF2R* are known to be involved in OS progression. *MAP3K5* also known as apoptosis signal-regulating kinase is a member of the MAPK pathway and activates *MAPK9* in response to various stress signals [66]. These genes have been reported to show context sensitive functions. On one hand, they are able to induce angiogenesis [67], and, on the other hand, they can induce apoptosis [68]. The cyclin dependent kinase inhibitor gene *CDKN2A* is involved in cell cycle regulation due to cyclin phosphorylation. Loss of function mutations or deletions of this gene can lead to continuous cell cycle progression. *CDKN2A* is frequently altered in various cancers [64] and also in OS [69]. Further, *EZR* is implicated in OS metastasis [51] and *IGF2R* is increased on the cell surface of OS cell lines [70].

**Table 5:** Genomic locations of potential prognostic genes in osteosarcoma.

Module	Entrez geneid	Symbol	Cytoband
1	4217	MAP3K	5q35
1	5601	MAPK9	6q22.33
2	7430	EZR	6q25.3
4	1915	EEF1A1	6q14.1
5	7337	UBE3A	15q11.2
7	28514	DLL1	6q27
7	102	ADAM10	15q22
15	6950	TCP1	6q25.3-q26
17	1029	CDKN2A	9p21
18	3482	IGF2R	6q26
20	311	ANXA11	10q23
23	257218	SHPRH	6q24.3

The table describes copy number associated genes with prognostic significance. For each gene, the module, the Entrez geneid, its official gene symbol, and its cytoband in the human genome (hg19) are given.

Contrary to that, the proliferation modules 3 and DNA damage module 6 are negative in our search for prognostic relevance and are not mentioned in publications to be of prognostic significance up to now. But this is now explainable by their module members. These modules contain the prominent OS-associated genes *RB1* and *TP53*. Patients with inherited diseases like Retinoblastoma or Li-Fraumeni syndrome possess mutations or alterations within the *RB1* and *TP53* genes, respectively, and are susceptible for OS development [4, 5]. Additionally, these genes are frequently affected in sporadic OS. Hence, it does not seem surprising that their biological network vicinity lacks prognostic significance, as the processes regulated by them or their interaction partners are constitutional for the tumorigenesis and tumor maintenance of OS. Thus the established scheme of functional modules for OS is well backed by prior knowledge and a stringent composition of the modules.

Looking on the genomic location of these genes in Table 5, it shows up that more than half of the factors with prognostic potential are located on chromosome 6q. Chromosome 6q shows frequent loss of heterozygosity in malignant tumors like breast cancer and ovarian cancer [71, 72]. Even in OS, a high allelic loss of several regions on chromosome 6q has been described [3, 73, 74] and could also be observed in this study. Nevertheless, the special role of chromosome 6q remains to be elucidated yet.

### Alternatives

Beyond the already cited screening studies, there are some more array based studies published [75] but none is following our comprehensive approach. Some studies are focusing solely on expression data and pathways or regulation aspects [76–78]. The latter studies are looking on downstream effects in the expressom itself. The very recent work from Both et al. [46], already mentioned in the GISTIC chapter, is centered on individual driver genes in OS. This approach is based on differential genes (human fetal osteoblast cell culture versus OS) and copy number data and therefore closer to our approach. Important differences exist by the source of the signals and the applied theoretical procedures. Nevertheless in all cases a direct comparison remains complicated because every approach possess his own work hypothesis and the results are heavily influenced by the choice of the methods.

Compared with Both et al. the integration of HPRD data and a dedicated network approach in our experimental design, in turn enables us to show how the genomic heterogeneity forms an intrinsic order on the level of the network modules.

## Conclusion

OS often present complex karyotypes including various copy number gains and losses, due to their high chromosomal instability. This complexity hampers the identification of a conceptual framework of OS biology. So if the order can not be established on the level of primary observations, it might be consequent to look at a higher level of abstraction, the biological network, especially the network modules. To address this issue, we adapted a network-based approach to formalize the complex pattern of factors, affected by individual genetic changes, using their functional associations within the cellular network.

The network approach showed several distinct modules with a specific functional context. The modules are further supported by well-described candidates of the pathogenesis of OS. These candidates are showing up in a consistent way in all modules according to their known functionality. Actually, candidates that were missing in our set of copy number associated genes appeared within the OS network as significant linker genes. These linker genes might be used further to deduce functional mechanisms for unknown candidate genes [79], for instance the putative prognostic genes detected on chromosome 6q.

To conclude, individual OS patients acquire different genomic alterations via diverse mechanisms that ultimately terminate in the typical clinical and morphological picture of OS [80]. Consequently, we observed a large genomic heterogeneity and complexity between individual patients. However, we illustrated that the different genomic aberrations all affect the same cellular network vicinity to maintain individual tumors.

## Supporting Information

S1 Fig Prognostic significance of ploidy patterns in osteosarcoma. The survival curve displays the survival frequency (y-axis) over time in months (x-axis). The OS samples were divided in non-diploid (ploidy > 3n, red) and diploid (blue) tumor samples. The prognostic significance was determined using the log-rank test.
(TIF)

S2 Fig Recurrent copy number variable regions. The genome-wide plot illustrates the frequency of copy number alterations, namely losses (green) and gains (red), across 41 OS biopsies. Frequencies are presented among the human chromosomes 1 to 22 (hg19). The dotted vertical line (gray) marks the 20% threshold of recurrent copy number alterations.
(TIF)

S3 Fig Copy number associated gene correlation. The density curves display the frequency (y-axis) of the Pearson correlation coefficients (x-axis) for the total number of genes on the Human Gene 1ST array (Affymetrix Inc., gray), the genes located within regions of recurrent copy number alterations defined by a frequency of 20% (blue), and genes located within regions of significant copy number alterations defined by GISTIC 2.0 (red). The correlation distributions were compared to each other using the Kolmogorov-Smirnov test.
(TIF)

S4 Fig Connectivity of the osteosarcoma network. The figures demonstrate frequency (y-axis) of **(A)** the number of interactions (x-axis) and **(B)** genes (x-axis) of random networks derived from the HPRD. The horizontal lines (red) indicate the observed value of the osteosarcoma network and the respective p-values.
(TIF)

S5 Fig Node degree distribution of the osteosarcoma network. The plot shows the fraction of genes (y-axis) among all node degrees (x-axis) of all genes within the osteosarcoma networks (gray). The horizontal lines indicate the average node degree of all genes (blue) and the degree threshold for hub genes (red). Hubs are defined as the top 5% of genes with highest degree.
(TIF)

S6 Fig Modularity of the osteosarcoma network. The plot displays the frequency (y-axis) among 1,000 modularity scores of random networks. The horizontal line (red) marks the observed modularity score of the OS network and lists its respective p-value.
(TIF)

S7 Fig Functional associations of members in the proliferation module 3. The network is derived from the STRING 9.0 database [65]. It illustrates experimental and literature-mined functional associations between genes within the proliferation module 3 of the osteosarcoma network.
(TIF)

S8 Fig Prognostic significance of copy number associated genes. The survival curves show the overall survival frequencies (y-axis) over time in months (x-axis). The OS samples were divided in copy number lost (green) and neutral (gray) tumor samples. The specific gene(s) analyzed regarding their prognostic significance are marked above the respective survival curves. The prognostic significance was determined using the log-rank test.
(TIF)

S1 Table Significant genomic alterations defined by GISTIC 2.0. The table reports all detected significant GISTIC regions. It lists the cytobands, peak coordinates, number of genes located within the respective regions, and the defined q-values.
(XLS)

S2 Table Key values to TP53, CDKN1A, or CDK4. The table reports **(A)** expression values, **(B)** copy number results by ASCAT and GISTIC of the three molecular factors.
(XLS)

S3 Table Cytoband information to Fig 3. The cytoband information of all genes in module 1, 3, 7 is given.
(XLS)
