# Supplementary figures and images for: Genomic Heterogeneity of Osteosarcoma - Shift from Single Candidates to Functional Modules
Source: PLoS One. 2015 Apr 7;10(4):e0123082. doi: 10.1371/journal.pone.0123082 (PMC4388529; doi:10.1371/journal.pone.0123082)

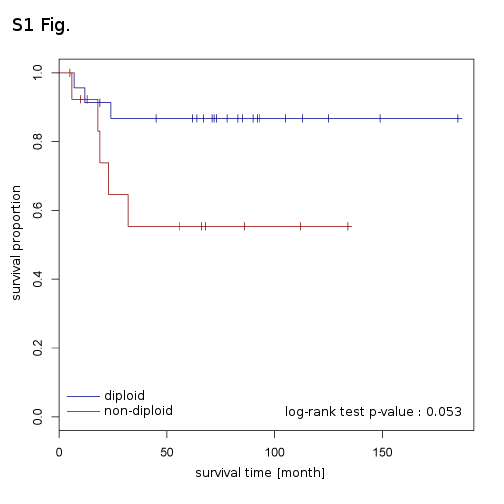

Supplement: S1 Fig — The survival curve displays the survival frequency (y-axis) over time in months (x-axis). The OS samples were divided in non-diploid (ploidy > 3n, red) and diploid (blue) tumor samples. The prognostic significance was determined using the log-rank test. (TIF) [file pone.0123082.s001.tif]

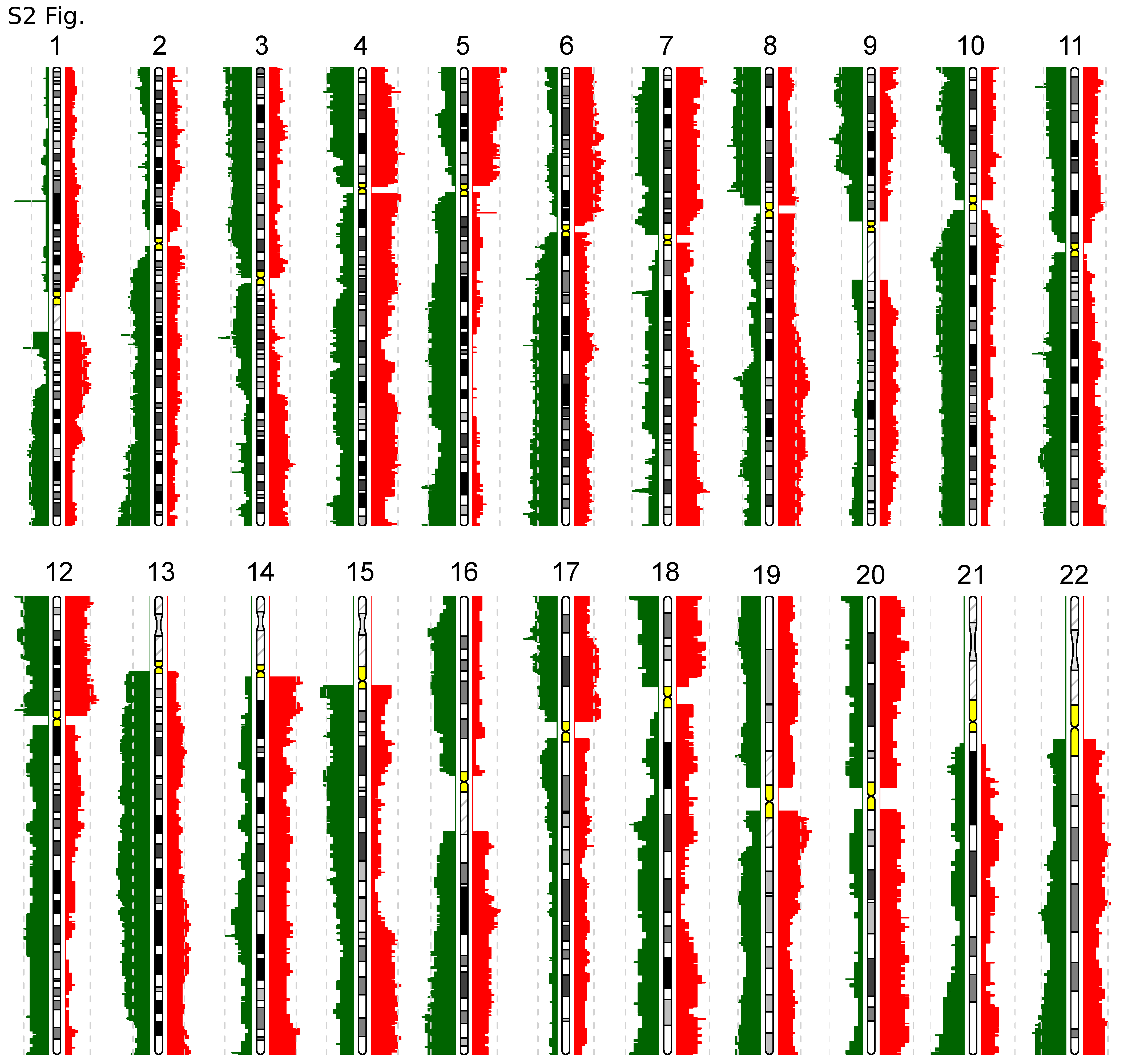

Supplement: S2 Fig — The genome-wide plot illustrates the frequency of copy number alterations, namely losses (green) and gains (red), across 41 OS biopsies. Frequencies are presented among the human chromosomes 1 to 22 (hg19). The dotted vertical line (gray) marks the 20% threshold of recurrent copy number alterations. (TIF) [file pone.0123082.s002.tif]

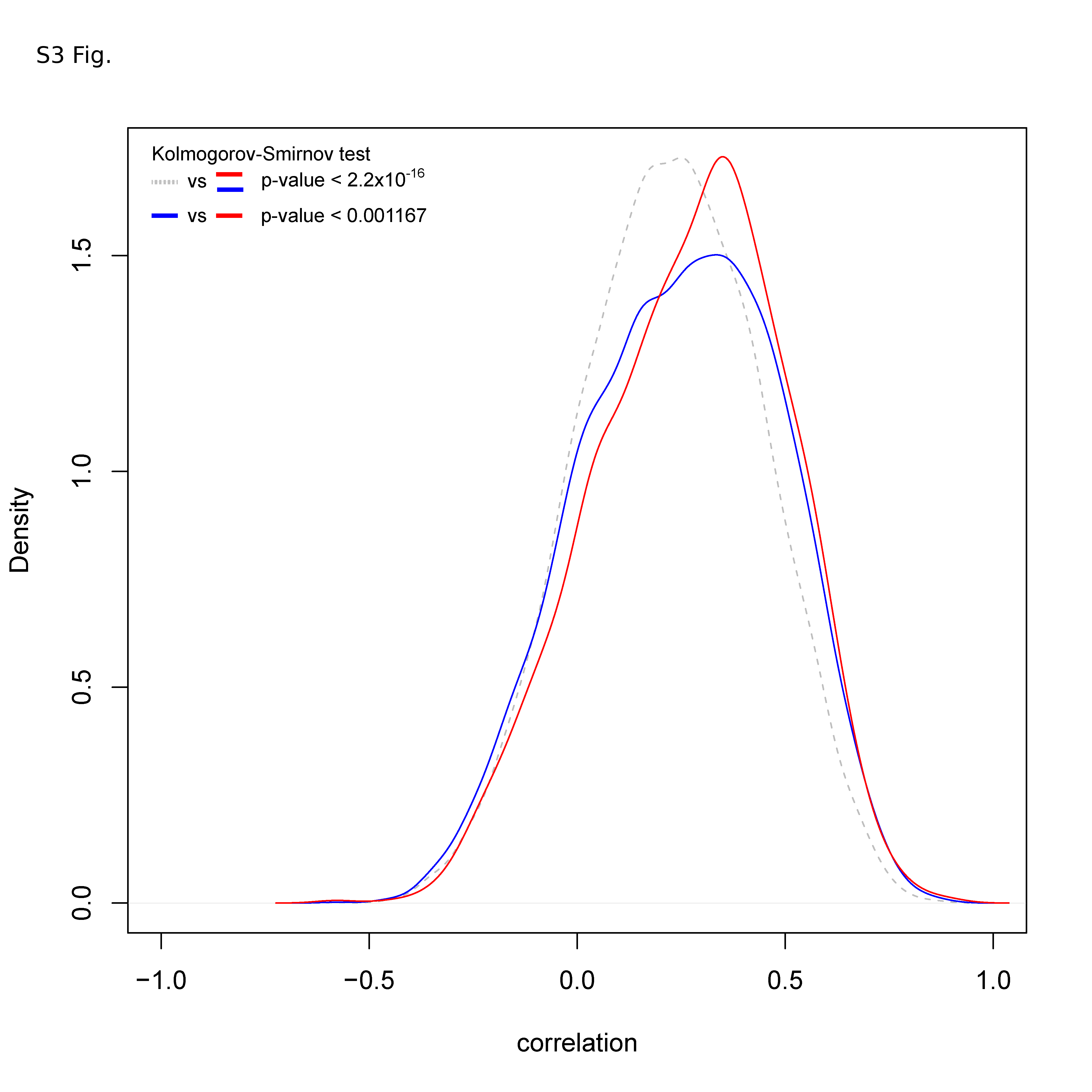

Supplement: S3 Fig — The density curves display the frequency (y-axis) of the Pearson correlation coefficients (x-axis) for the total number of genes on the Human Gene 1ST array (Affymetrix Inc., gray), the genes located within regions of recurrent copy number alterations defined by a frequency of 20% (blue), and genes located within regions of significant copy number alterations defined by GISTIC 2.0 (red). The correlation distributions were compared to each other using the Kolmogorov-Smirnov test. (TIF) [file pone.0123082.s003.tif]

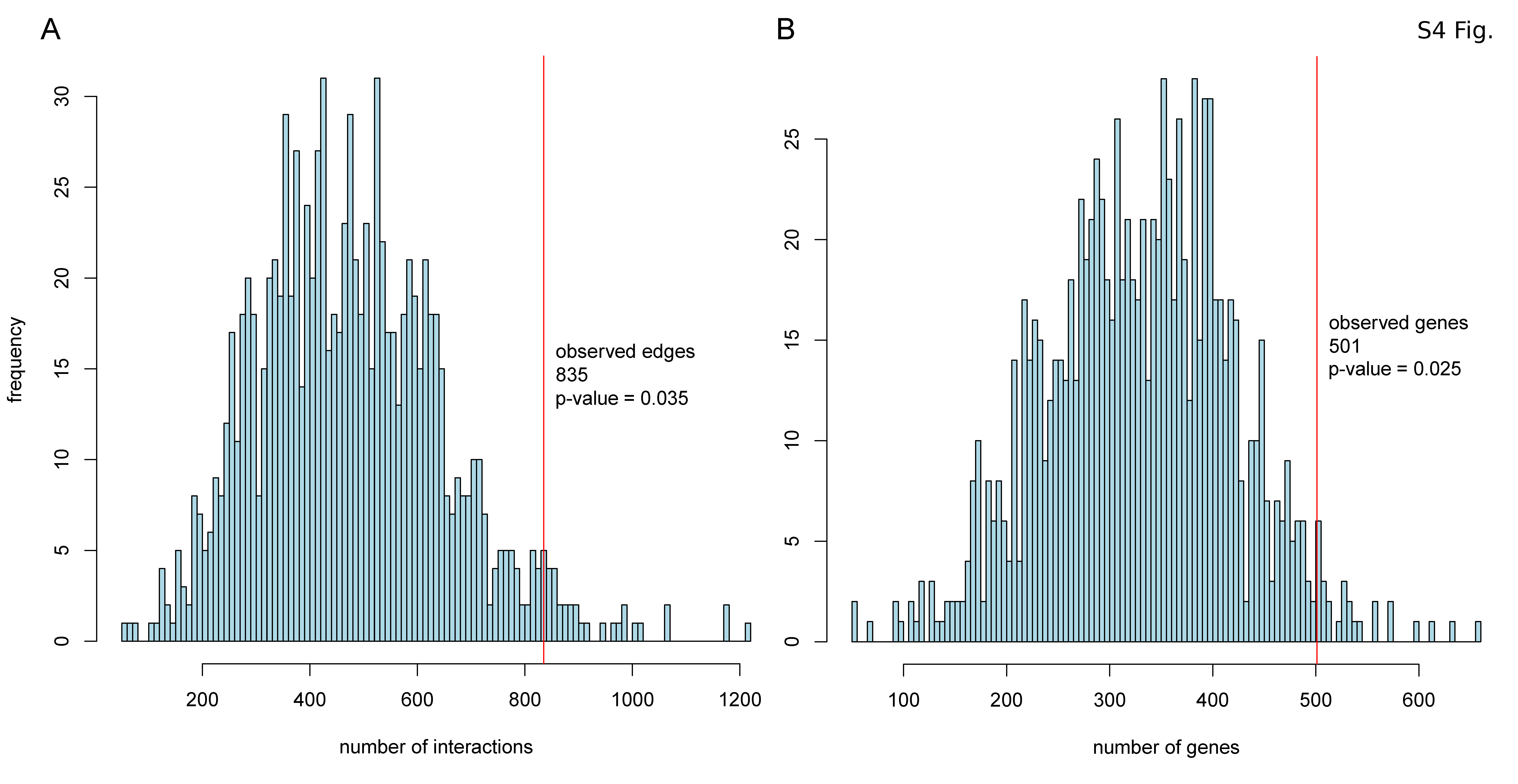

Supplement: S4 Fig — The figures demonstrate frequency (y-axis) of (A) the number of interactions (x-axis) and (B) genes (x-axis) of random networks derived from the HPRD. The horizontal lines (red) indicate the observed value of the osteosarcoma network and the respective p-values. (TIF) [file pone.0123082.s004.tif]

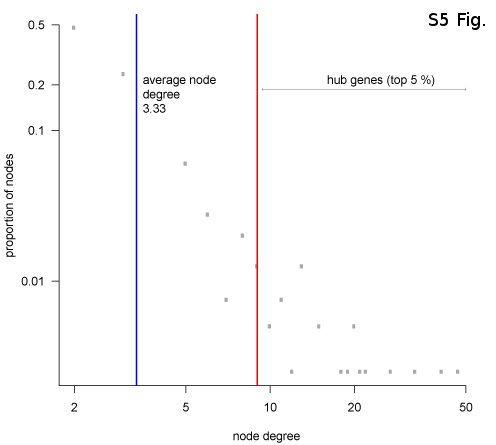

Supplement: S5 Fig — The plot shows the fraction of genes (y-axis) among all node degrees (x-axis) of all genes within the osteosarcoma networks (gray). The horizontal lines indicate the average node degree of all genes (blue) and the degree threshold for hub genes (red). Hubs are defined as the top 5% of genes with highest degree. (TIF) [file pone.0123082.s005.tif]

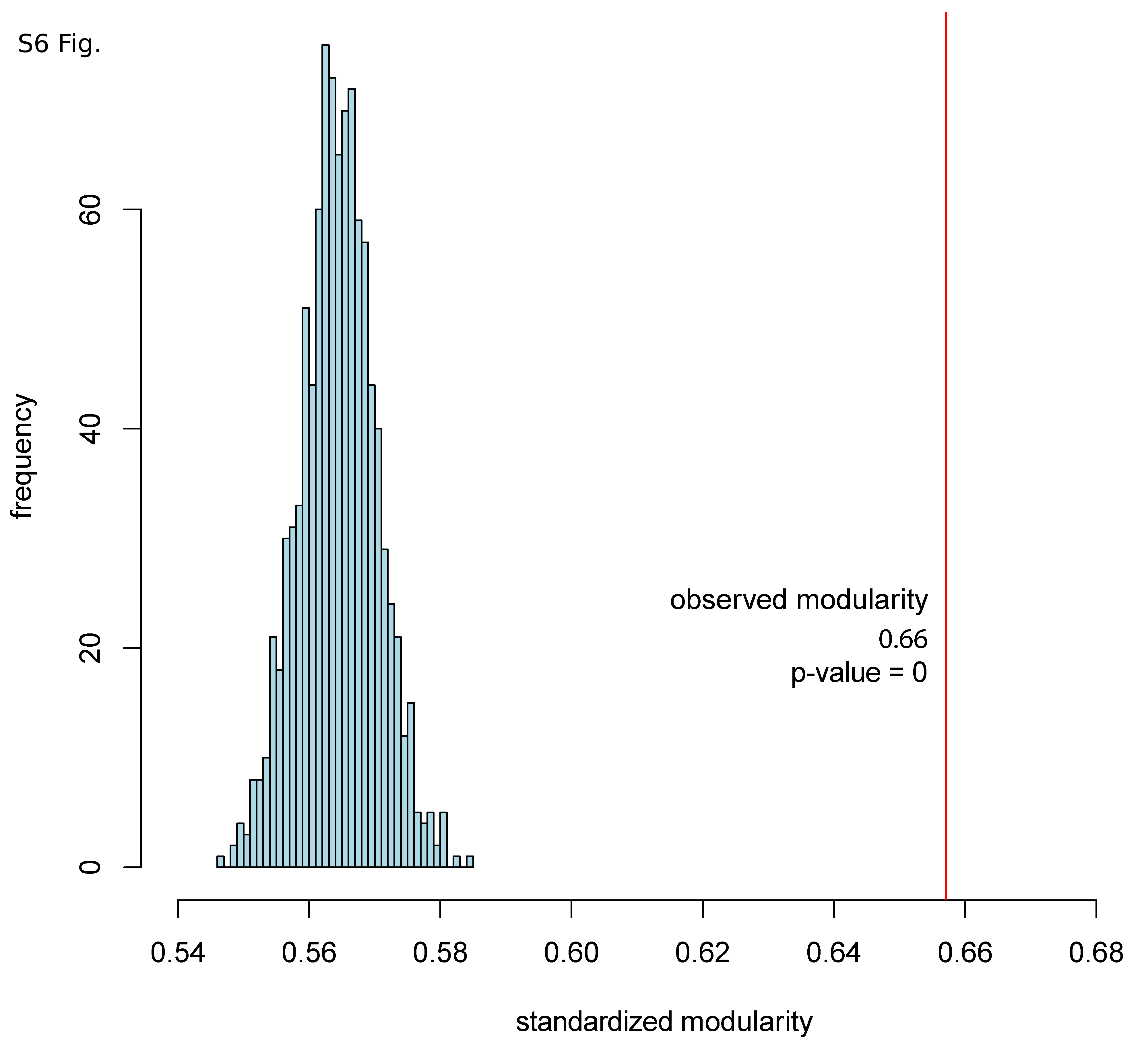

Supplement: S6 Fig — The plot displays the frequency (y-axis) among 1,000 modularity scores of random networks. The horizontal line (red) marks the observed modularity score of the OS network and lists its respective p-value. (TIF) [file pone.0123082.s006.tif]

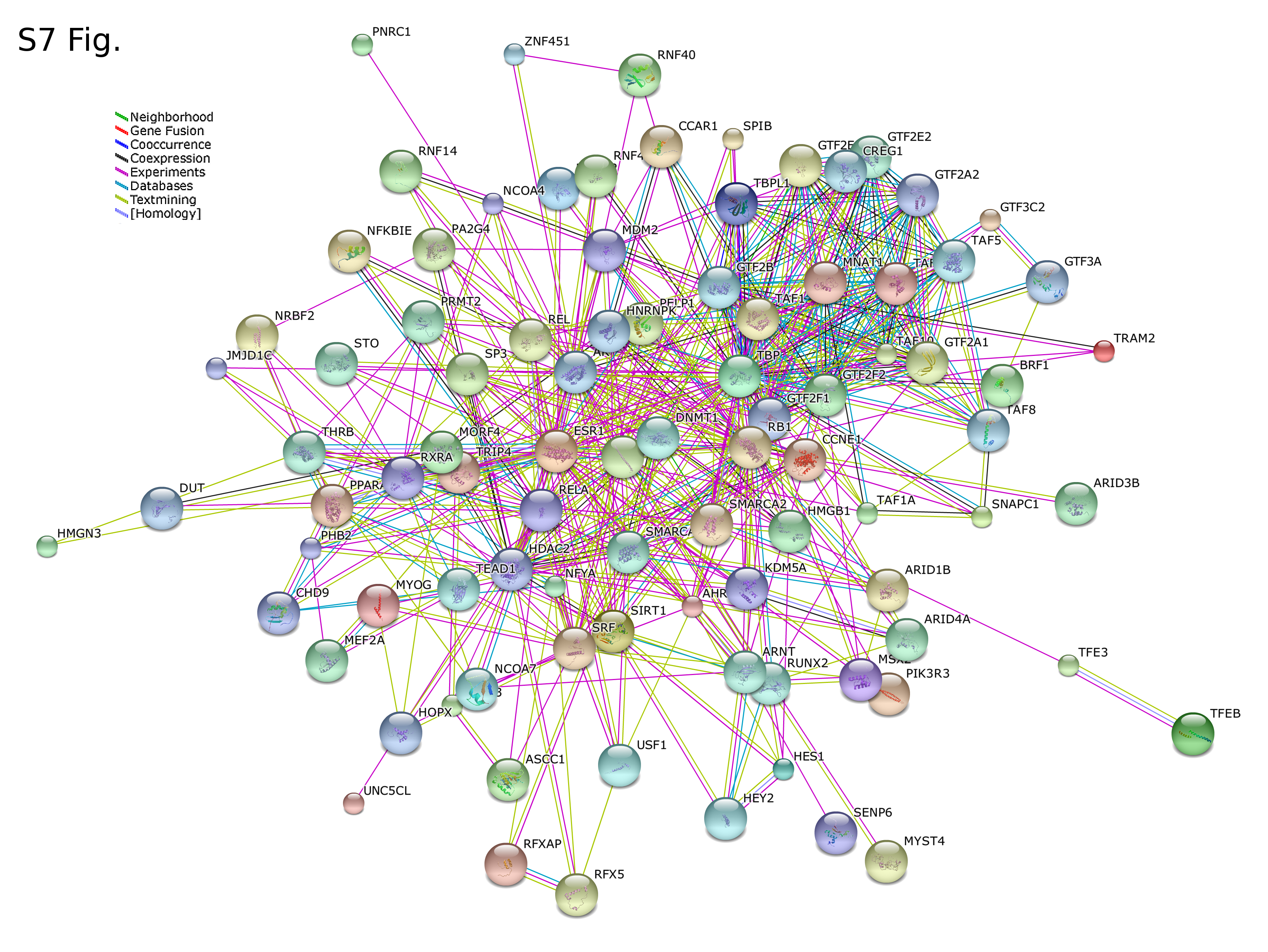

Supplement: S7 Fig — The network is derived from the STRING 9.0 database [65]. It illustrates experimental and literature-mined functional associations between genes within the proliferation module 3 of the osteosarcoma network. (TIF) [file pone.0123082.s007.tif]

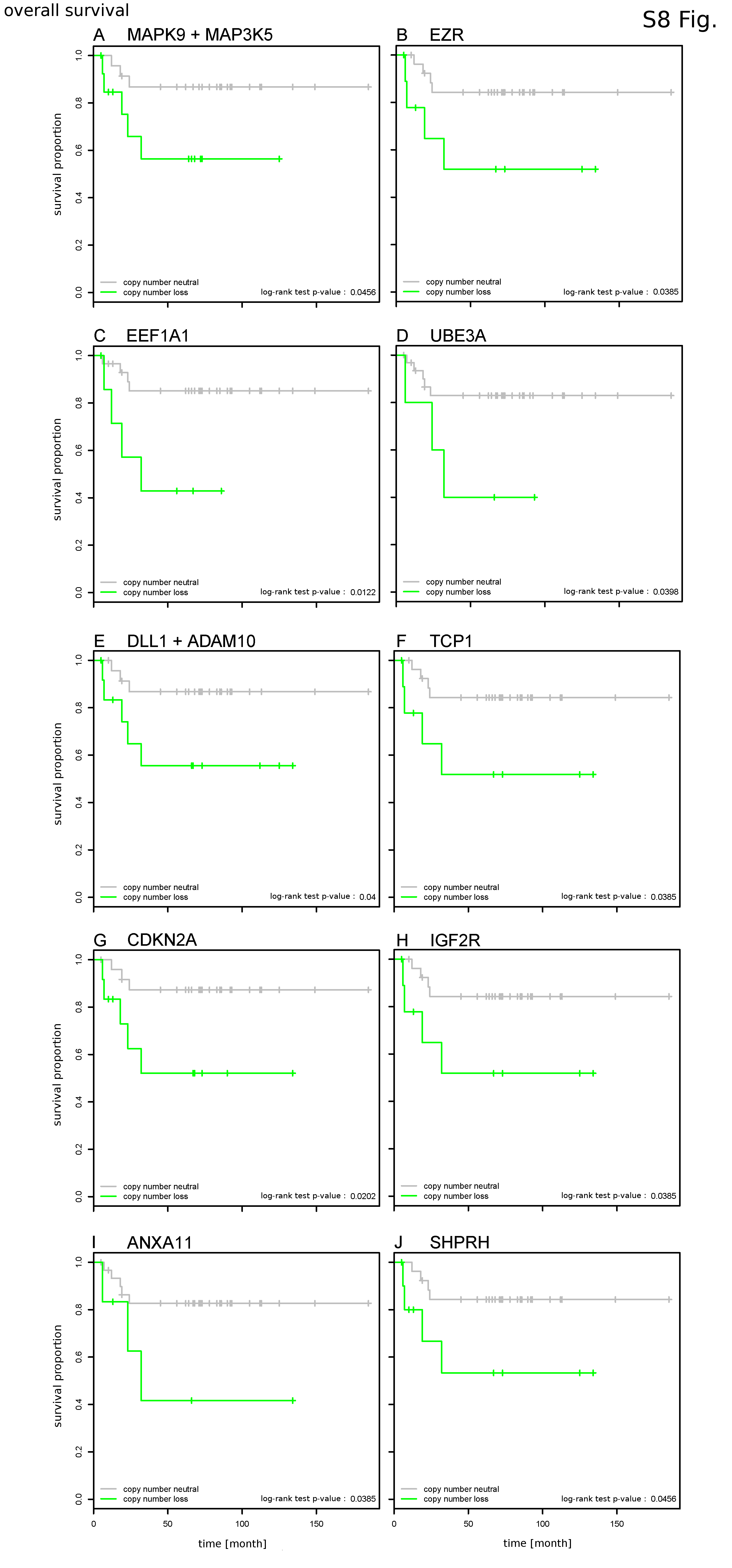

Supplement: S8 Fig — The survival curves show the overall survival frequencies (y-axis) over time in months (x-axis). The OS samples were divided in copy number lost (green) and neutral (gray) tumor samples. The specific gene(s) analyzed regarding their prognostic significance are marked above the respective survival curves. The prognostic significance was determined using the log-rank test. (TIF) [file pone.0123082.s008.tif]
